# Protein undernutrition alters the colonic bacteriome, disrupts intestinal immune homeostasis, and impairs control of *Leishmania infantum* infection in a murine model of visceral leishmaniasis

**DOI:** 10.3389/fnut.2025.1733703

**Published:** 2026-01-30

**Authors:** Renata Azevedo, Monica Losada-Barragán, Erika M. Costa, Heidi Pauer, Cristiane Cassiolato Pires Hardoim, Filipe Lima, Nathalia Pinho, Jonathan Durães, Felipe Gaitán-Albarracín, Sergio Cuervo-Escobar, Sebastián Arcila-Barrera, Adriana Umaña-Pérez, Luis Caetano M. Antunes, Patricia Cuervo

**Affiliations:** 1 Laboratório de Pesquisa em Leishmanioses, Instituto Oswaldo Cruz, Fiocruz, Rio de Janeiro, Brazil; 2 Grupo de Investigación en Biología Celular y Funcional e Ingeniería de Biomoléculas, Universidad Antonio Nariño, Bogotá, Colombia; 3 Laboratório de Bacteriologia Aplicada à Saúde Única e Resistência Antimicrobiana, Instituto Oswaldo Cruz, Fiocruz, Rio de Janeiro, Brazil; 4 Instituto Nacional de Ciência e Tecnologia de Inovação em Doenças de Populações Negligenciadas, Centro de Desenvolvimento Tecnológico em Saúde, Fundação Oswaldo Cruz, Rio de Janeiro, Brazil; 5 Department of Molecular Biosciences, University of Kansas, Lawrence, KS, United States; 6 Departamento de Fisiologia, Instituto de Biociências, Universidade de São Paulo, São Paulo, Brazil; 7 Departamento de Química, Facultad de Ciencias, Grupo de Investigación en Hormonas, Universidad Nacional de Colombia, Sede Bogotá, Bogotá, Colombia

**Keywords:** colon, dysbiosis, IL-13, *Leishmania infantum*, microbiota, undernutrition, visceral leishmaniasis

## Abstract

**Introduction:**

Undernutrition is a significant global health issue that exacerbates susceptibility to infectious diseases, including visceral leishmaniasis (VL), caused by *Leishmania infantum*. Here, we investigated the interplay between undernutrition, immune responses, and the colonic microbiota composition in a murine model of VL.

**Methods:**

We used BALB/c mice subjected to a low-protein diet and infected with *L. infantum* to analyze the effects on systemic and local immune responses, microbiota composition, and parasite load.

**Results:**

Undernutrition significantly downregulated the mRNA expression of cytokines such as IFN-γ, IL-10, IL-12, and IL-17A in the colon while increasing the colonic luminal levels of proinflammatory cytokines, including TNF-α, CCL5, and IL-17A. The protein-deficient diet also induced dysbiosis, characterized by reduced *Bacteroidota* and increased *Desulfobacterota* and *Firmicutes*. Additionally, secretory IgA levels were markedly elevated in undernourished animals, suggesting a compensatory response to dysbiosis. The parasite load was significantly increased in the spleens of undernourished, infected mice, potentially due to disrupted immune-endocrine communication involving intestinal inflammation and microbial imbalance.

**Discussion:**

These findings highlight the complex interplay between nutritional status, the microbiota, and host immunity in the progression of VL. Undernutrition exacerbates disease severity through local and systemic immune dysregulation and microbial shifts. Our results support new treatments targeting diet and microbiota to control VL.

## 1 Introduction

Undernutrition is a significant public health problem that affects millions of people worldwide, including both children and adults. Immunity or susceptibility to infectious parasitic diseases is directly related to the host’s nutritional status (1, 2). Protein-energy undernutrition is one of the main determinants of increased susceptibility to infectious diseases and is a known risk factor for developing clinical manifestations and severe forms of visceral leishmaniasis (VL) (3–5).

Visceral leishmaniasis (VL) is caused by intracellular protozoan parasites of the *Leishmania* genus, specifically *L. donovani* and *L. infantum*. Visceral leishmaniasis, caused in the Americas and the Mediterranean basin by the intracellular protozoan *Leishmania infantum* (syn. *L. chagasi*), is a life-threatening disease endemic in developing regions where poverty, malnutrition, and weak health infrastructures converge (6, 7). The clinical outcome of *L. infantum* infection is highly variable, ranging from asymptomatic carriage to fatal systemic disease. This heterogeneity is strongly influenced by host factors, with protein-energy undernutrition being a well-established key determinant of increased susceptibility, severity, and mortality (2–4). Undernutrition is thought to compromise innate and adaptive immune mechanisms necessary for parasite control, thereby exacerbating disease progression. Early experimental studies using murine models of polynutrient undernutrition and VL caused by *L. donovani* demonstrated an early increase in parasite burden in the liver and spleen of undernourished mice. This increase was associated with a compromised lymph node barrier function, elevated prostaglandin E_2_ (PGE_2_) levels, and altered cellular trafficking (4, 8–10).

Our group established a murine model focused on low-protein consumption and infection with *L. infantum*. This model revealed a significant reduction in T-cell subpopulations in the thymus and spleen of undernourished mice, along with a quiescent thymic and splenic microenvironment characterized by significantly decreased cell proliferation (11–14). Furthermore, we observed that the disorganization of the splenic microarchitecture caused by the parasite was aggravated when preceded by protein-undernutrition, including a decrease in the number of germinal centers. Proteomic analysis of the spleen further demonstrated alterations in the abundance of secreted proteins as early as 15 days post-infection. These changes were either diminished or heightened in animals subjected to undernutrition before infection. Specifically, we observed shifts in the abundance of proteins involved in cell migration and immune responses in the spleens of undernourished-infected mice (11, 12, 15). These changes were accompanied by an early and significant increase in the splenic parasite load in undernourished animals, suggesting that protein undernutrition may alter the cell-mediated immune response in the spleen of animals infected with *L. infantum*, thereby accelerating or aggravating the clinical course of VL (11, 14).

In our model, we also observed inflammatory infiltrates in the duodenum and colon of well-nourished-infected mice, which were aggravated when undernutrition preceded infection (14). Infection with *L. infantum* also induced a significant increase in duodenal immunoglobulin A (IgA) in well-nourished animals, but these levels were significantly decreased in undernourished-infected mice. Additionally, we detected elevated levels of Th17-related cytokines in the duodenum of undernourished animals, corroborating local inflammation (16). These findings suggest that *L. infantum* infection is significantly sensed in the intestine, and that the gut microbiota may play a potential role in the response to the parasite.

Intestinal microbial composition is influenced by dietary habits, just as the immune system depends on microbial metabolism (17). Healthy intestinal microbiota is essential for maintaining host health and performs protective, structural, and metabolic functions that directly and indirectly impact nutrition (17, 18). Based on our previous results, we hypothesize that dysbiotic events in undernourished mice, occurring before parasite infection, impair both local and systemic responses to *L. infantum* infection. To test this hypothesis, we explored how protein undernutrition modulates immune responses and colonic microbial composition in a murine model of *L. infantum* infection. By integrating nutritional status, immune markers, and microbiota profiling, we demonstrate that a low-protein diet exacerbates intestinal inflammation, induces dysbiosis, and impairs the host’s ability to control splenic parasite burden. These findings underscore the complex interplay between diet, gut homeostasis, and systemic immunity in the context of VL. Such integrative approaches are essential to advancing our understanding of how nutritional interventions may influence the outcome of infectious diseases, particularly in populations where undernutrition and parasitic infections frequently coexist.

## 2 Materials and methods

### 2.1 Ethics statement

This study was conducted under the guidelines outlined in the *Guide for the Care and Use of Laboratory Animals* (Eighth Edition), published by the National Institutes of Health. Animal procedures were approved by the Instituto Oswaldo Cruz Animal Care and Use Committee (License CEUA-IOC, No. 038/2018-A1). The *L. infantum* strain employed in this research, MCAN/BR/2000/CNV-FEROZ, was supplied by the *Leishmania* Collection of the Instituto Oswaldo Cruz, Rio de Janeiro (CLIOC).^1^ In compliance with Brazilian Biodiversity Law, this study was registered with SisGen (AA2236F).

### 2.2 Parasite culture

Parasites were cultured at 25°C in Schneider’s medium supplemented with 10% fetal bovine serum (FBS). They were harvested and collected at the stationary phase by centrifugation at 1,800 × *g* for 5 min and then washed twice in PBS (pH 7.20).

### 2.3 Animals, dietary regimen, and *L. infantum* infection

The BALB/c undernutrition model was developed according to previous reports (16). Male mice aged 3–4 weeks, with an initial body weight of 12–16.5 g ± 2.0 g, were housed under a 12-h light/dark cycle in ventilated racks with individually ventilated mini-isolator cages equipped with filtered air supply and exhaust. The room temperature was maintained between 20 and 26°C, with relative humidity ranging from 40 to 60%. The cages, designed for mice, measure 207 mm in height, 216 mm in width, and 316 mm in length (total floor area of 451 cm^2^), housed a maximum of five animals. Animals had free access to water, and food was provided as described as follows. Briefly, 3-week-old male BALB/c mice (*n* = 48), were fed a standard diet containing 14% protein (MP Biomedicals, Inc., United States, Catalog No. 960258). After 7 days, 24 mice were maintained on the 14% protein diet, whereas the remaining 24 were switched to a 4% protein diet (MP Biomedicals, Inc., United States, Catalog No. 960254). The two provided diets were isocaloric, and animals had access to water and food *ad libitum*. After 1 week on different diets, animals were further subdivided: one subgroup was infected with 1 × 10^7^ *L. infantum* parasites via intravenous injection into the caudal vein, whereas the other group received saline solution as a control. This resulted in four experimental groups: (1) animals fed the 14% protein diet (control protein, CP) (*n* = 12), (2) animals fed the 4% protein diet (low protein, LP) (*n* = 12), (3) animals fed the 14% protein diet and infected with *L. infantum* (CPi) (*n* = 12), and (4) animals fed the 4% protein diet and infected with *L. infantum* (LPi) (*n* = 12). Animals were fed their respective diets postinfection, for the remainder of the experiment. They were also followed daily after infection, and their body weights were recorded every 3 days. At 14 days postinfection, the animals were euthanized. According to the National Council for the Control of Animal Experimentation of Brazil (CONCEA) Euthanasia Guidelines, euthanasia was performed by an overdose of sodium thiopental at a dose of 300 mg/kg, preceded by pre-anesthetic medication consisting of 10% ketamine hydrochloride (200 mg/kg) and 2% xylazine hydrochloride (10 mg/kg), administered intraperitoneally. Both procedures were performed using 1.0 mL syringes and 26G 1/2 needles (13 × 0.45 mm). Euthanasia was considered effective when there was no response to painful stimuli, and there was clinical confirmation of the absence of heartbeat and respiration. Once the absence of a heartbeat had been verified using a stethoscope—or, following cervical dislocation as a secondary method—euthanasia was deemed complete. Blood was collected via cardiac puncture, and serum was separated and stored at −30°C. The spleen and liver were removed, weighed, and used for nucleic acid extraction. Parasite loads in the liver and spleen of BALB/c mice infected with *L. infantum* were measured using qPCR, following previously established protocols (19). The intestine was sectioned to obtain the colon and gently washed with PBS to collect luminal contents, which were filtered through a 100-μm membrane to remove insoluble particles and centrifuged at 5,000 × *g*. The resulting supernatant (luminal fluid) and pellet were separated and stored at −80°C.

### 2.4 Cytokine expression analysis

Total RNA was isolated from the colon using TRIzol reagent according to the manufacturer’s instructions (Life Technologies, Inc.). RNA concentration was measured with a Nanodrop ND-1000 spectrophotometer (NanoDrop Technologies), and cDNA was synthesized from 1 μg of total RNA using the SuperScript III reverse transcription system (Invitrogen). Real-time quantitative PCR was performed using SYBR^®^ Green PCR Master Mix on a ViiA7 system (Applied Biosystems), following the supplier’s guidelines. The analyzed genes were: *il-10*, *il-12*, *ifn-*γ, *tnf-*α, *tgf-*β, and *il-17a*, as well as the reference genes *hprt*, *atp-*β*5*, and *cyc-1* (Supplementary Material 1). PCR conditions and relative expression analysis were performed as previously described (12). Data are presented as normalized ratios between the expression of the target gene and the geometric mean of the three reference genes. qPCR was performed following the MIQE guidelines.

### 2.5 Cytokine levels

Levels of CCL5, CXCL9, CXCL10, IFNγ, IL-7, IL-12, IL-13, IL-17A, IL-21, IL-22, IL-23, TGFβ, and TNFα in the colon luminal fluid were measured using a multiplex immunoassay with fluorescence-encoded beads, following the manufacturer’s guidelines (BioLegend). Data acquisition was conducted using a BD FACSCanto II flow cytometer, and subsequent analysis was carried out with LEGENDplex software (version 8.0). Each assay was performed in duplicate and results are presented as pg/mL.

### 2.6 Secreted immunoglobulin A (sIgA) levels

Secreted IgA levels in the colonic luminal fluid were quantified using an ELISA, following the manufacturer’s instructions (Invitrogen Catalog No. 88-50450).

### 2.7 Microbiota composition analyses

To analyze the bacterial composition of the colon microbiota, animals were euthanized and the contents of their colon were immediately collected. DNA was extracted using the QIAamp DNA Stool Minikit (Qiagen, Germany) with modifications. Mechanical lysis was performed in PowerBead Tubes, Garnet (Qiagen, Germany), by mixing the samples with 1 mL of InhibitEX buffer and incubating at 95°C for 5 min. Tubes were then placed horizontally in a vortex adapter and vortexed at maximum speed for 10 min. Then, the tubes were centrifuged at 13,000 × *g* for 1 min, and the supernatant was transferred to a clean 2 mL tube. The remaining steps were carried out according to the manufacturer’s instructions, and the purified DNA was stored at −80°C until further analysis.

Sequencing of the gene encoding 16S rRNA was performed using standard Illumina protocols. First, variable regions V3 and V4 of the 16S rRNA gene were amplified using primers 5’- CCTACGGGNGGCWGCAG-3’ and 5’-GACTACHVGGGTATCTAATCC-3’ and KAPA HiFi HotStart ReadyMix enzyme (Roche, United States). After preparation, libraries were sequenced on a Illumina MiSeq system with 500 cycles of chemistry v2. Sequencing was performed at the *Plataforma de Sequenciamento de Ácidos Nucleicos de Nova Geração*—RPT01J of *Fundação Oswaldo Cruz* (Rio de Janeiro, Brazil).

Illumina reads were processed using Mothur v. 1.44 (20). The pipeline is described in detail in Hardoim et al. (21). Briefly, paired raw reads were quality checked and reduced to unique sequences. The alignment was performed using the SILVA seed v. 138 database (mothur-formatted), provided by Mothur, as reference (22, 23). Non-redundant sequences were maintained in the dataset and preclustered. UCHIME (24) was implemented to identify chimeric sequences, which were removed from the dataset. Then, sequences were phylogenetically classified and undesirable (mitochondria, chloroplast, Eukaryota, and unknown) and singletons were removed from the dataset. The remaining sequences were assigned to operational taxonomic units (OTUs) at 97% sequence similarity. The resulting OTU table was normalized to 8434 sequences per sample. In total, 261,454 reads were further analyzed. Each OTU was classified based on the SILVA non-redundant v. 138 database (mothur-formatted) (22, 23). The generated OTU table was then processed using the MicrobiomeAnalyst online software suite^2^ in Marker Data Profiling mode. All sequences generated in this study were deposited in the Sequence Read Archive in the NCBI database with Bioproject ID: PRJNA1262530.^3^

### 2.8 Statistical analyses

Statistical analysis was conducted using GraphPad Prism 8.0 and MicrobiomeAnalyst software. Two-way analysis of variance (ANOVA) followed by Tukey’s *post-hoc* test was applied to assess differences between treatments. Alpha diversity data were analyzed using the Kruskal-Wallis test with multiple comparison FDR correction with the Benjamini, Krieger and Yekutiley test. Beta diversity was analyzed with using the Bray-Curtis index as the distance method and PERMANOVA. Significant differences (*p* < 0.05) due to diet, infection, or their interaction are indicated by the letters a, b, or c, respectively.

## 3 Results

### 3.1 Undernourished BALB/c mice infected with *L. infantum* present decreased body, spleen and thymus weight, and diminished IGF1 serum levels

**FIGURE 1:**
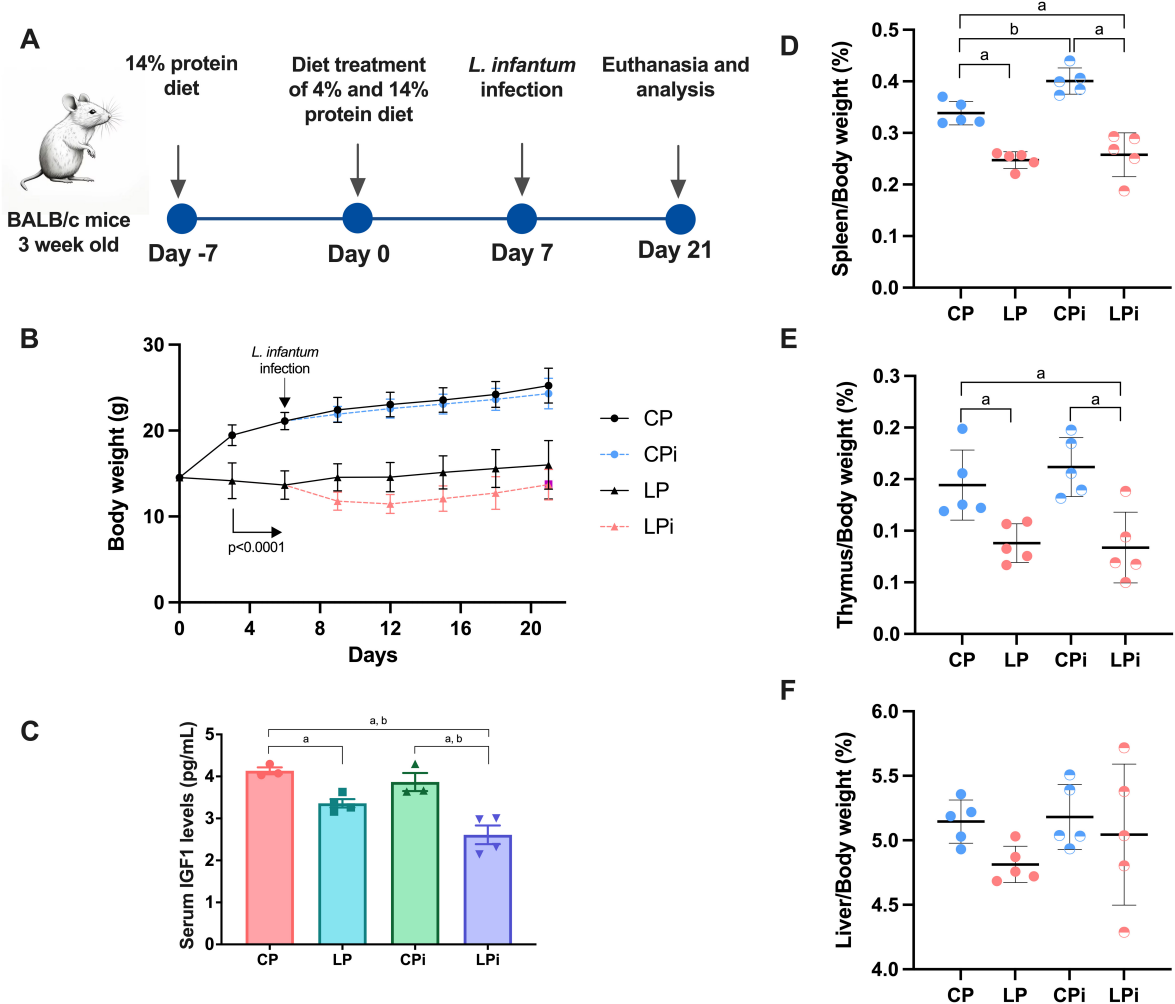
Experimental design and effect of protein malnutrition on body and tissue weight in mice infected with *L. infantum*. **(A)** Schematic representation of the experimental design. BALB/c male mice were fed a 14% (*n* = 24, CP) or 4% (*n* = 24, LP) protein diet for 21 days. On day 7 of the experimental period, half the animals were infected with *L. infantum* and the other half received a saline solution injection. CP: animals fed 14% protein diet; LP: animals fed 4% protein diet, CPi: animals fed 14% protein diet and infected; LPi: animals fed 4% protein diet and infected. Two weeks later, the animals were euthanized and assessed for susceptibility to infection, immunological parameters and bacteriome analysis. **(B)** Body weight was registered every third or 4 days and expressed as average ± SEM; *n* = 12 mice in each group. Statistical differences before the day of infection were determined by Student’s *t*-test (*p* < 0.001). Total IGF1 **(C)** serum levels were measured by specific murine ELISA immunoassays. **(D)** Spleen, **(E)** thymus and **(F)** liver weight gain at day 21 expressed as a percentage of tissue/body weight ± SEM (*n* = 12). Statistical differences due to (a) diet (*p* < 0.0001), (b) infection (*p* < 0.05), were determined by two-way ANOVA.

Male BALB/c mice were maintained on isocaloric diets containing either normal (14%) or low (4%) protein levels for 21 days. On the seventh day, the animals were randomly split, and half were infected with *L. infantum*, creating four experimental groups: CP (control protein, 14% diet), LP (low protein, 4% diet), CPi (control protein and infected), and LPi (low protein and infected). The animals were euthanized 14 days after infection (Figure 1A). In agreement with our previous reports (11–14, 16), mice body weight showed significant divergence as early as day three of the dietary intervention (*p* < 0.0001) (Figure 1B). Since protein deficiency typically leads to reduced serum levels of IGF1, this hormone was quantified. After 21 days on the low-protein diet, IGF1 concentrations were markedly lower in the undernourished (LP and LPi) groups (*p* = 0.0002) compared to well-nourished mice due to diet or to *L. infantum* infection (*p* = 0.0150) (Figure 1C).

In LP mice, a reduction in the relative weight of lymphoid organs was also noted. Specifically, the spleen (*p* < 0.0001) and thymus (*p* < 0.0001) showed significant weight decreases when compared to CP mice after 21 days on the low-protein diet (Figures 1D,E). In contrast, CPi exhibited an increase in spleen weight following parasite infection (*p* = 0.0116) (Figure 1D). However, malnourished infected mice (LPi) did not show such increase, suggesting that protein deficiency impairs lymphoid organ responses to infection. Liver weight was unchanged in all experimental groups (Figure 1F).

### 3.2 Undernutrition reduces mRNA levels of cytokines IFNγ, IL-10, IL-12, and IL-17A in the colon of BALB/c mice infected with *L. infantum*

**FIGURE 2:**
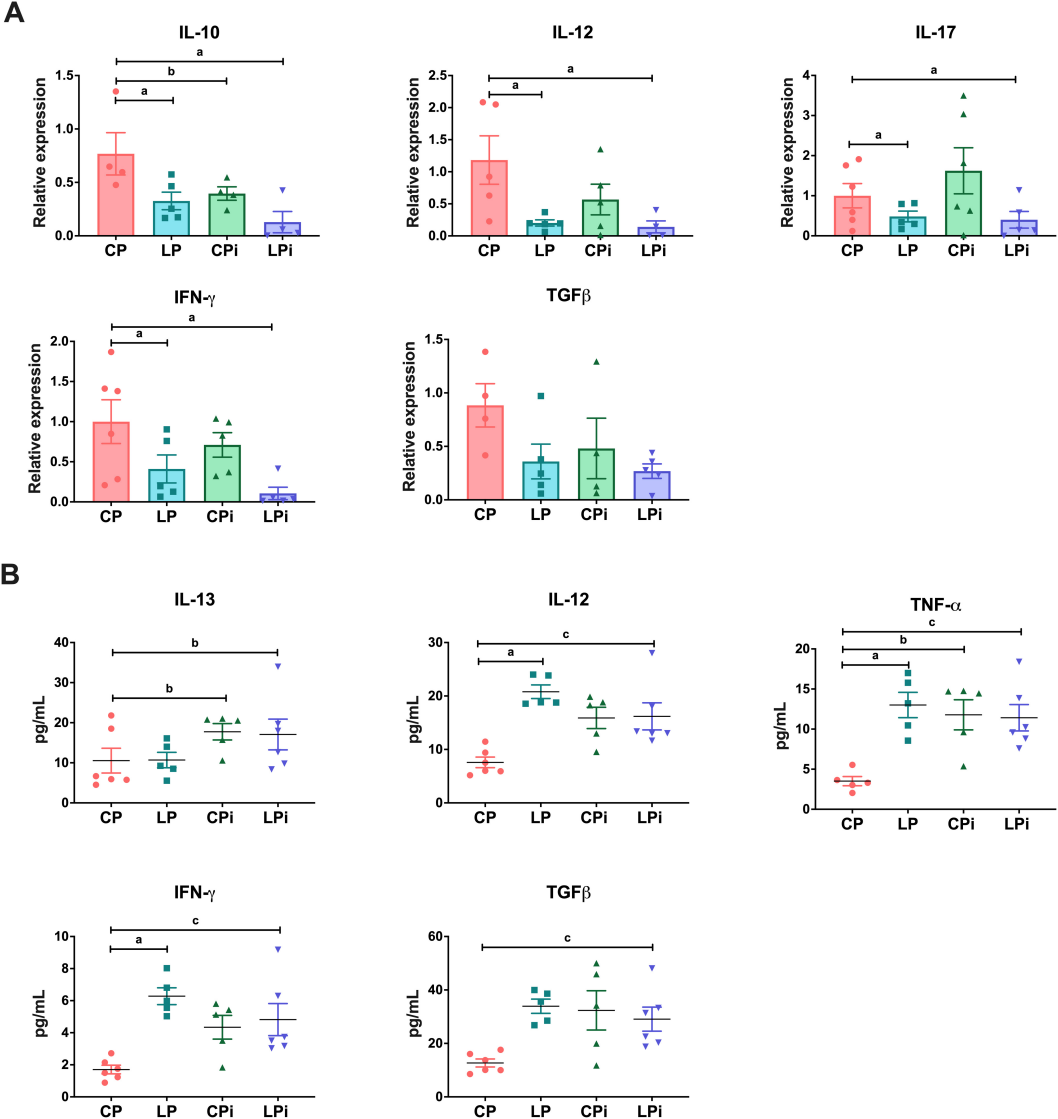
Cytokine transcripts and protein levels expression in the colon of undernourished BALB/c mice infected with *L. infantum.* **(A)** *IL-10, IL-12, IL-17*, *IFN-*γ, and *TGF-*β mRNA expression levels in the colons of each experimental group were measured by qPCR. The values are expressed as normalized ratios between the expression levels of the target gene and the geometric median of the *hprt*, *atp-*β*5*, and *cyc-1* genes. Statistical differences due to (a) diet (*p* < 0.001), (b) infection (*p* < 0.05), or (c) the interaction between diet and infection (*p* < 0.05) were determined by two-way ANOVA. **(B)** The IFNγ, IL-12, IL-13, TGFβ, and TNF-α, protein levels in the colonic luminal fluid at 14 days post-infection were measured by flow cytometry using a multiplex assay. Statistical differences due to (a) diet (*p* < 0.05), (b) infection (*p* < 0.05), or (c) the interaction between diet and infection (*p* < 0.05) were determined by two-way ANOVA. CP, animals fed a 14% protein diet; LP, animals fed a 4% protein diet, CPi, animals fed a 14% protein diet and infected; LPi, animals fed a 4% protein diet and infected.

As we previously reported in our murine model, the colon was significantly affected by undernutrition, showing histopathological alterations, including lymphocytic inflammatory infiltrates in the lamina propria and submucosa, as well as infiltration by neutrophils, macrophages, and plasma cells (16). Therefore, we assessed whether undernutrition could alter cytokine gene expression in this intestine segment in both non-infected and *L. infantum*-infected mice. Our results showed that undernutrition significantly reduced the expression of IFNγ (*p* = 0.0075), IL-10 (*p* = 0.0105), IL-12 (*p* = 0.0109), and IL-17A (*p* = 0.0329) in the colon, regardless of parasite infection status (Figure 2A). In the case of IL-10, there was also a significant decrease in expression when well-nourished (CP) animals were infected with *L. infantum* (*p* = 0.0319). In contrast, TGFβ transcript levels did not significantly differ between treatment groups (Figure 2A). Additionally, TNFα mRNA levels were undetectable under all experimental conditions.

### 3.3 Both undernutrition and *L. infantum* infection increase protein levels of cytokines IFN-γ, IL-12, IL-17A, TGFβ, and TNF-α in the colons of BALB/c mice

At the protein level, we evaluated the impact of undernutrition and *L. infantum* infection on cytokine abundance in the luminal fluid of the colon using a flow cytometry-based multiplex assay. Our analysis revealed that the levels of IFN-γ (*p* = 0.0093), IL-12 (*p* = 0.0025), TGFβ (*p* = 0.0121), and TNF-α (*p* = 0.0050) were significantly increased in the colons of LPi mice, resulting from the interaction of both conditions: low-protein diet and infection (Figure 2B). Notably, IFN-γ (*p* = 0.0021), IL-12 (*p* = 0.0018), and TNF-α (*p* = 0.0084) were also significantly elevated by the low-protein (LP) diet in the absence of infection, while TNF-α levels (*p* = 0.0434) were also influenced by infection, which increased their abundance in CPi animals. Furthermore, IL-13 levels were significantly higher in both the CPi and LPi groups due to *L. infantum* infection (*p* = 0.0362).

**FIGURE 3:**
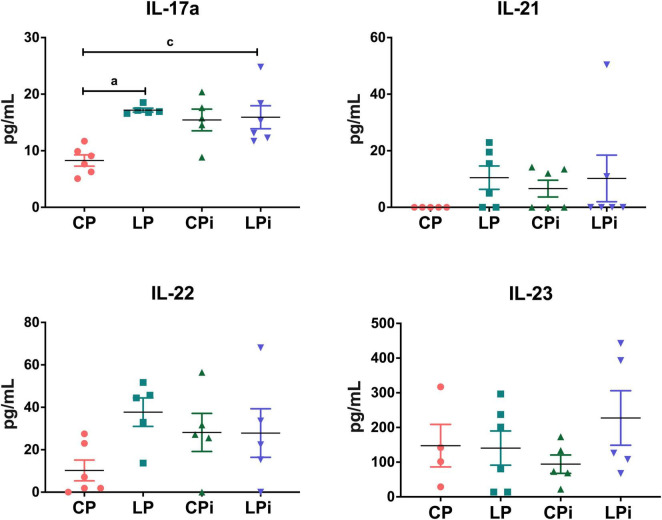
Th17-related cytokines levels in the colonic luminal fluid of undernourished mice infected with *L. infantum*. IL-17A, IL-21, IL-22, and IL-23 protein levels in the colonic luminal fluid at 14 days post-infection were measured by flow cytometry using a multiplex assay. Statistical differences due to (a) diet (*p* < 0.05), (b) infection (*p* < 0.05), or (c) the interaction between diet and infection (*p* < 0.05) were determined by two-way ANOVA. CP, animals fed a 14% protein diet; LP, animals fed a 4% protein diet; CPi, animals fed a 14% protein diet and infected; LPi, animals fed a 4% protein diet and infected.

Interestingly, the colons of undernourished mice presented elevated protein levels of IL-17A (*p* = 0.0066), a key cytokine associated with the Th17 immune response (Figure 3). Notably, in the LPi group, IL-17A levels were significantly increased due to the combined effects of the LP diet and *L. infantum* infection (*p* = 0.0130). However, the levels of IL-21, IL-22, and IL-23 were similar across all experimental groups.

### 3.4 CCL5 protein levels were significantly increased, whereas CXCL9 was reduced in the colons of undernourished animals

**FIGURE 4:**
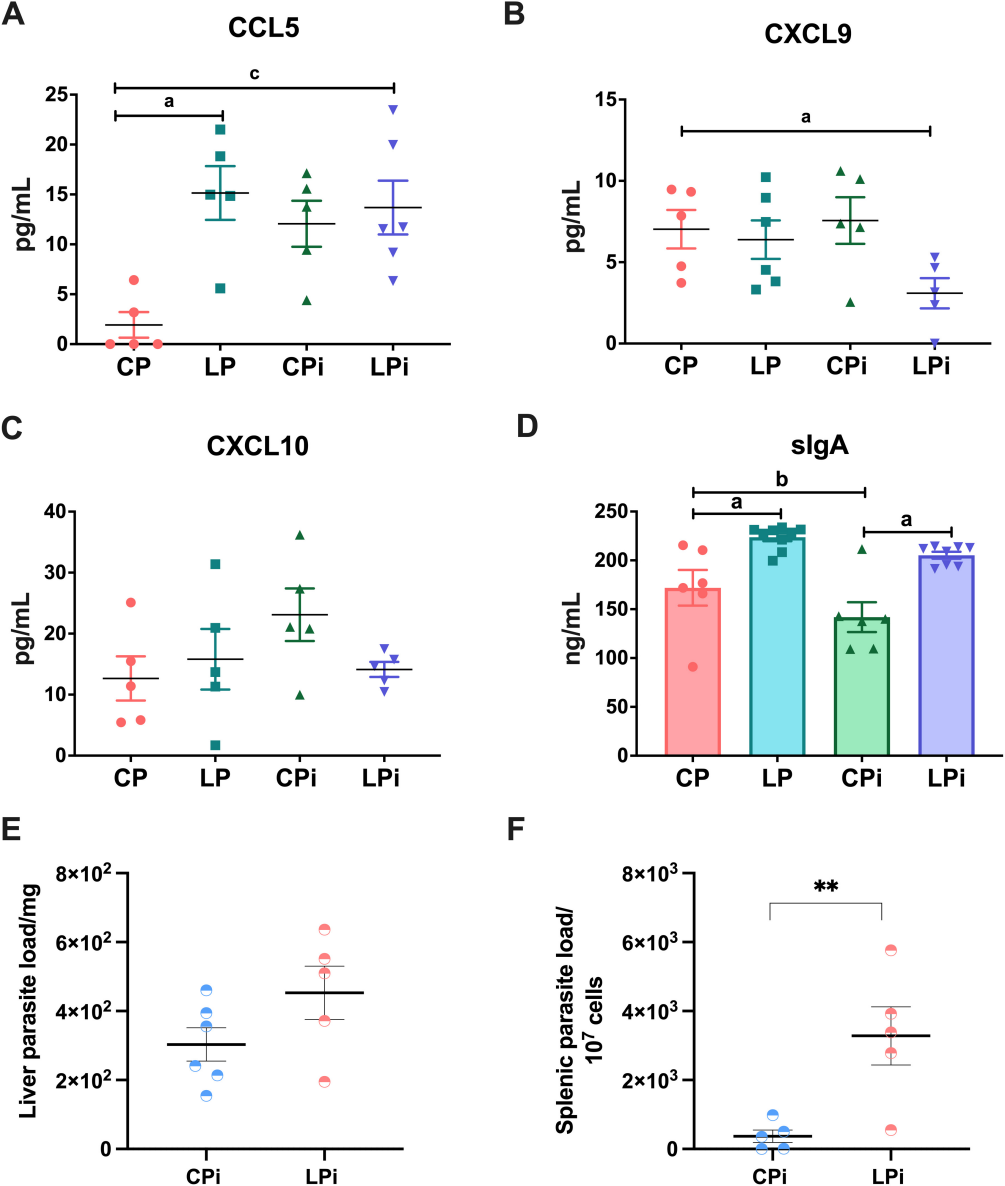
Chemokine, sIgA levels and parasite load in undernourished BALB/c mice infected with *L. infantum*. **(A)** CCL5, **(B)** CXCL9, and **(C)** CXCL10 levels in the colonic luminal fluid at 14 days post-infection were measured by flow cytometry using a multiplex assay whereas **(D)** sIgA levels were measured by ELISA. Statistical differences due to (a) diet (colon *p* < 0.01), (b) infection (*p* < 0.01), or (c) the interaction between diet and infection (*p* < 0.05) were determined by two-way ANOVA. **(E)** Liver and **(F)** spleen parasite loads were measured by qPCR using the TaqMan^®^ system in CPi and LPi animals. Statistical differences were determined with Student’s *t*-test (*p* = 0.0098). *n* = 5–6 mice per group. CP, animals fed 14% protein diet; LP, animals fed 4% protein diet; CPi, animals fed a 14% protein diet and infected; LPi, animals fed a 4% protein diet and infected.

The increase in IL-17 suggests an inflammatory microenvironment in the colon. Therefore, we analyzed the protein levels of chemokines involved in inflammatory responses and cell recruitment. We observed that they were significantly altered by undernutrition and by the interaction between the LP diet and *L. infantum* infection. Notably, there was a significant increase in CCL5 in the colon of LP mice due to undernutrition (*p* < 0.05), as well as in the colon of LPi mice due to the interaction between diet and infection (*p* = 0.0112) (Figure 4A). Furthermore, CXCL9 was significantly reduced in the colon of LPi animals due to undernutrition (*p* < 0.05) (Figure 4B). No significant changes were observed in the concentration of CXCL10 (Figure 4C).

### 3.5 Undernourished mice exhibit a significant increase in IgA levels in the colon

Since sIgA is primarily found in mucosal secretions, we hypothesized that its levels might be affected in the colon. To test this hypothesis, we measured the concentration of sIgA secreted into the colonic luminal fluid. Interestingly, the abundance of sIgA in the colon was altered primarily by the low-protein diet (Figure 4D). The colons of LP and LPi animals showed a significant increase in sIgA abundance due to undernutrition (*p* < 0.05) (Figure 4D). This increase represents a 1.5-fold higher IgA concentration in the colons of undernourished animals compared to control animals.

### 3.6 Undernourished mice exhibit increased splenic parasite loads

Liver and spleen samples were collected from malnourished or well-nourished mice infected with *L. infantum*. Parasites were detected in the liver and the spleen by a previously described qPCR assay (19). qPCR results showed that 100% of liver and spleen samples were positive for *L. infantum* (Figures 4E,F). Although not significantly different, parasite loads in the liver were higher in LPi animals compared to CPi animals (Figure 4E). Additionally, in agreement with our previous reports, there was a significant increase in parasite loads in the spleens of LPi mice at 14 dpi (*p* = 0.0098) (Figure 4F).

### 3.7 Undernutrition alters bacterial composition in the colon, increasing the relative abundance of *Desulfobacterota* and *Firmicutes* while reducing *Bacteroidota* abundance

**FIGURE 5:**
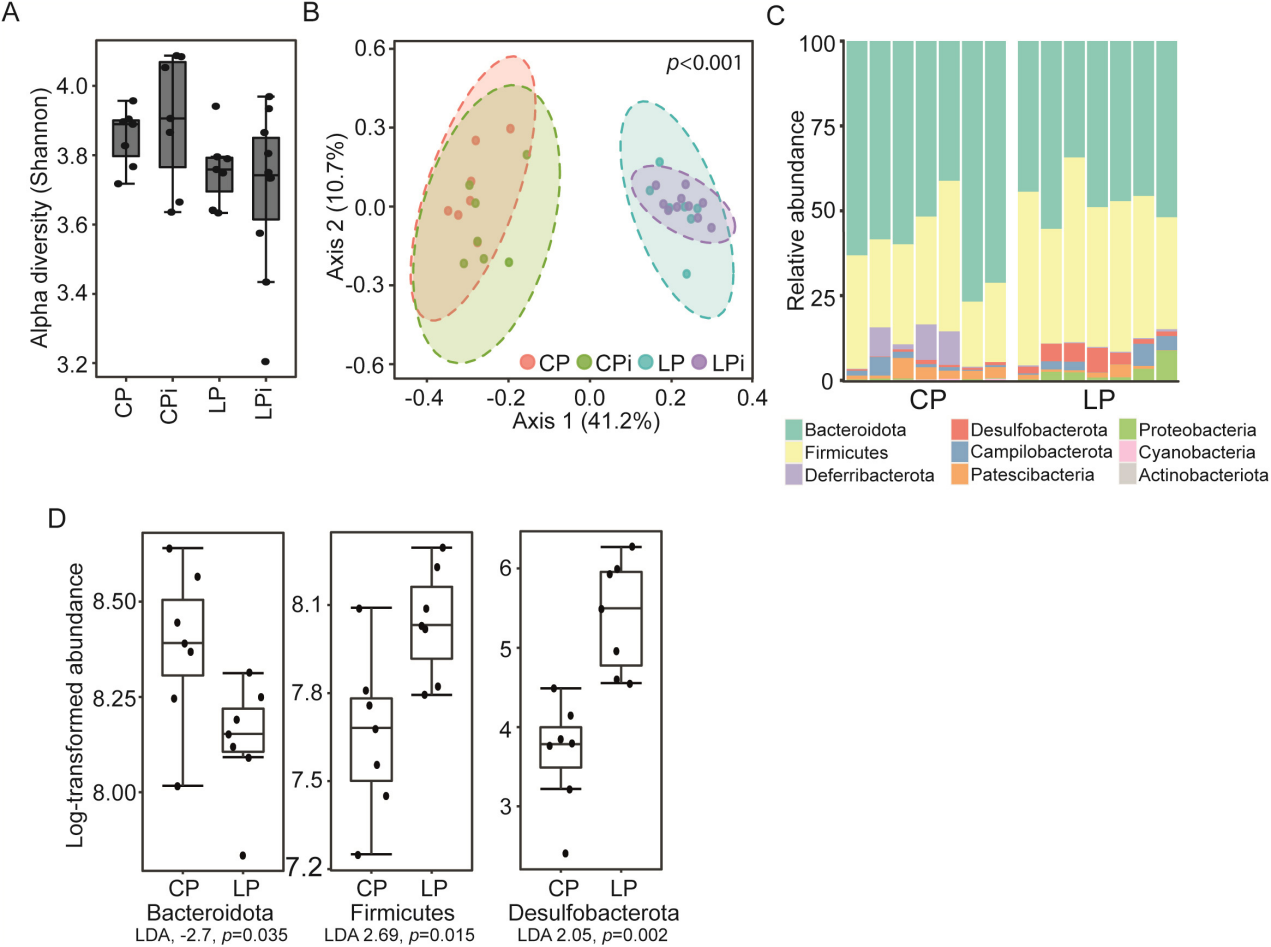
Effects of malnutrition and *Leishmania* infection on the colonic bacterial microbiota. **(A)** Alpha diversity of gut bacterial communities in animals fed a control diet or a low-protein diet with or without *Leishmania* infection. Each dot indicates one animal. No significant differences were observed between groups, as determined by ANOVA followed by Tukey’s multiple comparisons test. **(B)** Principal coordinate analysis (PCoA) showing the beta diversity of the bacteriome of animals fed different diets with or without infection. Each dot represents one animal. The groups were significantly different, as determined by PERMANOVA. **(C)** Bacterial composition, at the phylum level, of the gut microbiota of uninfected animals fed each of the diets used. Each vertical bar represents one animal. **(D)** LEfSe analysis results showing abundance levels for the three phyla with LDA scores > 2 and *p* < 0.05. Each dot represents one animal.

The observed changes in cytokine, chemokine, and IgA levels suggest an inflammatory milieu in the colon, which could be associated with dysbiosis. To test this hypothesis, we investigated whether the LP diet and *L. infantum* infection induced alterations in colonic bacterial composition. No significant differences in alpha diversity were found between the experimental groups (Figure 5A). However, PCoA analysis revealed significant differences in beta diversity between well-nourished and undernourished mice, with or without infection (*p* < 0.001) (Figure 5B).

Next, to determine whether undernutrition-related effects on beta diversity were associated with specific bacterial populations in the colon, we analyzed bacterial composition at the phylum level across experimental groups. We observed significant differences in the phyla composing of the intestinal bacteriome of undernourished animals, whether infected or not, compared to well-nourished mice (Figure 5C). Specifically, the colonic bacteriome of undernourished animals (LP and LPi) exhibited a significant decrease in the abundance of *Bacteroidota* members, compared to well-nourished animals (CP and CPi). Conversely, undernourished mice displayed increased abundance of *Firmicutes* and *Desulfobacterota* members (Figures 5C,D).

**FIGURE 6:**
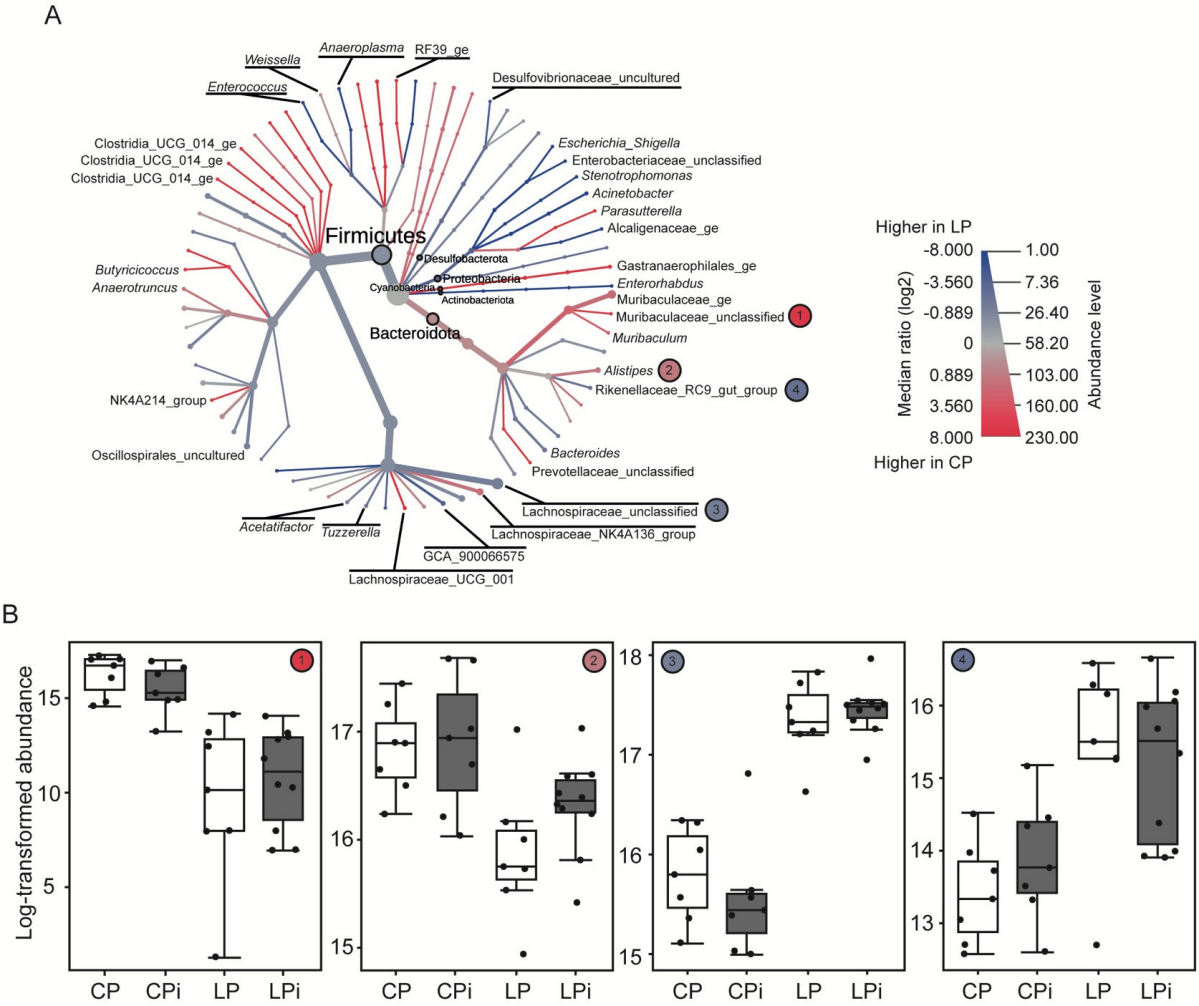
Taxa abundances in colons of malnourished mice infected with *L. infantum*. **(A)** Heat tree showing phyla and genera associated with each type of diet. The thickness of lines and nodes represents the abundance of taxa, whereas colors indicate which diet taxa are associated with. Red indicates higher levels in the bacteriome of animals fed the control diet; blue indicates higher levels in animals fed the low-protein diet. Phyla nodes are indicated by black circles. **(B)** Abundance levels of selected genera from E, as indicated by the circles with numbers. The two genera with the highest absolute LDA scores in each of the diet treatments were chosen. CP, animals fed a 14% protein diet; LP, animals fed a 4% protein diet; CPi, animals fed a 14% protein diet and infected; LPi, animals fed a 4% protein diet and infected.

The heat tree shown in Figure 6A compares the relative abundances of various taxa between the CP and LP diets. LEfSe analysis identified the most discriminative OTUs between the CP and LP groups (Figure 6B). A member of the *Alistipes* genus as well as a member of an unclassified genus within the *Muribaculaceae* family, were significantly reduced in undernourished mice. On the other hand, undernourished animals (LP and LPi) exhibited significantly higher abundance of a member of the *Rikenellaceae* RC9 gut group and a member of an unclassified genus within the *Lachnospiraceae* family. Together, these four taxa were the primary contributors to the distinct microbiome profile observed in undernourished mice (LP and LPi), compared to the well-nourished groups (CP and CPi).

## 4 Discussion

Although undernutrition is a well-established risk factor for severe clinical forms of VL, the direct causal relationship between diet, bacteriome, and the progression of *L. infantum* infection remains to be fully elucidated. In this study, we show that a low-protein diet leads to significant changes in the diversity and abundance of bacterial taxa in the colon of BALB/c mice, both in infected and uninfected animals. The significant shifts observed, including a decrease in *Bacteroidota* and an increase in *Desulfobacterota* and *Firmicutes* (*p* < 0.05), provide a concrete microbial signature of protein undernutrition. Although we did not find a direct causal link between undernutrition, the bacteriome, and *L. infantum* infection, it is plausible that these statistically significant changes in the bacteriome could indirectly influence immune responses to the parasite.

Remarkably, the relationship between nutrition and the bacteriome is bidirectional. Nutritional status influences bacteriome populations, which in turn modulate host functions (25). In this context, the dysbiosis observed in undernourished mice in our study supports previous findings that protein malnutrition disrupts the bacteriome, leading to chronic intestinal inflammation, impaired amino acid absorption, and diarrhea (25). Consistent with these findings, we observed severe histopathological changes in the intestines of animals fed a low-protein diet, suggesting nutrient malabsorption (16).

Furthermore, intestinal fluid from the colons of undernourished mice exhibited a statistically significant proinflammatory profile, with elevated levels of CCL5 (*p* < 0.05), IFN-γ (*p* = 0.0093), IL-12 (*p* = 0.0025), IL-17A (*p* = 0.0066), and TNF-α (*p* = 0.0050). This specific inflammatory milieu, characterized by the interplay of Th1 (IFN-γ, TNF-α) and Th17 (IL-17A) cytokines alongside the chemokine CCL5, is crucial for our hypothesis. It likely contributes directly to leukocyte recruitment, immune dysregulation, epithelial damage, and intestinal barrier disruption, all of which negatively affect nutrient absorption (26). Indeed, leukocyte recruitment and epithelial damage were observed histologically (16). A compromised intestinal barrier also facilitates the passage of harmful substances and bacterial translocation from the gastrointestinal tract to extraintestinal sites, triggering systemic inflammation (27), thus, perpetuating the cycle of inflammation and disease. This local breakdown of barrier integrity provides a plausible pathway for the systemic immune dysfunction that culminates in the failure to control splenic parasite burden.

The dysbiosis observed in undernourished animals may also help explain the significantly increased levels of secretory IgA (sIgA) in their colon (*p* < 0.05, a ∼1.5-fold increase). Maintaining intestinal homeostasis requires a delicate balance of sIgA; imbalances in this molecule are associated with dysbiosis (28, 29). sIgA plays a crucial role in regulating the bacteriome by promoting the clearance of pathobionts and controlling the abundance of commensal bacteria (30, 31). Interestingly, sIgA also enhances intestinal immune competence and alleviates intestinal inflammation in undernourished mice (32). Thus, the increased sIgA levels observed in the colon of undernourished mice suggest a robust but likely insufficient local, functional adaptive response to dysbiosis. While this response may be critical for host antimicrobial defense and bacteriome recovery, its occurrence alongside significant inflammation indicates it fails to fully restore homeostasis or prevent the systemic consequences of undernutrition.

Studies examining the impact of low-protein diets on the gut bacteriome, particularly in mice and humans, remain limited. Factors such as diet type (caloric, carbohydrate, protein and fat-restricted), age, and the specific populations studied can significantly influence bacteriome composition. Most research on the gut bacteriome in nutrition has focused on obese models, which typically show an increase in *Firmicutes* and a decrease in *Bacteroidota*, the two dominant bacterial phyla of the human and murine gut microbiomes (33–35). Paradoxically, we observed a similar bacteriome profile in undernourished animals. Some studies suggest that *Firmicutes* are particularly efficient at extracting energy from undigested food, thus contributing to more efficient caloric extraction (36). The bacteriome can rapidly adapt to increase energy extraction from the diet, promoting energy storage in the host (37). In our model, this bacterial remodeling may represent an adaptive response to a low-protein diet, optimizing energy utilization under nutrient-limited conditions. The statistical increase in the *Firmicutes/Bacteroidota* ratio (*p* < 0.05) is therefore not merely a compositional note but a potential indicator of a microbiome in an energy-scavenging state, which may inadvertently sustain an inflammatory environment.

Notably, we detected a significant increase in *Desulfobacteria* in undernourished animals (*p* < 0.05). Many species in this phylum rely on sulfate reduction as their primary energy source. Sulfate-reducing bacteria are commonly found in the human gut, particularly in the colon, and have been linked with inflammatory bowel disease (38). While sulfate-reducing bacteria are often associated with inflammation (38, 39), hydrogen sulfide (H_2_S) produced by these bacteria has both pro- and anti-inflammatory effects, depending on its concentration (40–42). At low concentrations, H_2_S has beneficial effects, whereas high concentrations can be toxic and increase intestinal permeability (43). The significant increased abundance of *Desulfobacteria* in our study likely reflects a microbial adaptation to the low-protein diet and may be a direct contributor to the inflammatory profile (e.g., elevated TNF-α, IL-17A) observed in undernourished and infected animals. However, further studies are needed to better define the microbial lineages within this phylum and their specific contributions to inflammation.

Our findings also suggest that undernutrition significantly reduces the abundance of several taxa in the colon of BALB/c mice, including members of the *Alistipes* genus and the *Muribaculaceae* family (LEfSe analysis, LDA score > 2). This reduction may impair the production of signals necessary for inducing antimicrobial peptides (44, 45). Additionally, tryptophan deficiency in protein-free diets can lead to vitamin B3 deficiency, which further reduces the production of epithelial antimicrobial peptides (25). The loss of these antimicrobial peptides disrupts the colonic bacteriome and, in combination with epithelial damage, results in a destructive inflammatory response. Inflammatory states have been shown to favor the growth of *Enterobacteriaceae* (26), and we observed an increase in this bacterial family in undernourished animals, which was correlated with histopathological changes (16) and elevated inflammatory cytokines. We hypothesized that in our undernourished model, dysbiosis may also be influenced by tryptophan deficiency from a low-protein diet, further reducing antimicrobial signals.

IL-13, secreted in the intestine by various innate immune cells, primarily Th2 CD4^+^ T cells, plays a key role in responding to allergies, parasitic infections, and chronic inflammation. This cytokine targets multiple cell types, including epithelial cells, macrophages, smooth muscle cells, and neurons (46, 47). Regarding dietary conditions in our study, while a low-protein diet does not alter IL-13 levels in the colon, evidence suggests that gut-derived IL-13 promotes hepatic IGF-1 production (48), which could influence both growth and immune cell production. However, our current and previous findings indicate a reduction in hepatic and serum IGF-1 levels in undernourished animals [this study *p* = 0.0002, Figure 1C, and (12)], suggesting impaired communication between the gut and liver, which could impact immunocompetence under a low-protein diet.

Conversely, in the context of infection, although no parasites were detected in the colons of the animals, as previously reported (16), we observed a significant increase in IL-13 levels in response to parasitic infection in both undernourished and well-nourished animals (*p* = 0.0362). During enteric parasitic infections, IL-13 is typically associated with immune defense, intestinal barrier recovery, mucus production (which indirectly affects bacterial communities) (49), and modulation of intestinal motility (50, 51). Thus, its increase in our model suggests an immune-endocrine communication between the intestine and *L. infantum*-infected tissues, potentially serving as a protective signal for maintaining intestinal barrier integrity. The fact that this increase occurs even in the absence of *L. infantum* detection in the gut highlights a systemic sensing of the infection.

Additionally, in our model, intestinal damage in undernourished mice was associated with a significantly higher parasite burden in the spleen (*p* = 0.0098), suggesting a potential endocrine connection between the intestine and spleen that may influence parasite control. The cascade initiated by diet-induced dysbiosis and inflammation (statistically supported by changes in microbiota and cytokines) appears to undermine systemic immunity, evidenced by the failure to increase spleen weight post-infection in LPi mice (unlike CPi mice, *p* = 0.0116) and the consequent higher parasite load. Further studies are needed to confirm these hypotheses and fully explore the potential endocrine pathways linking the spleen, liver, and intestine during *L. infantum* infection.

In summary, our study demonstrates that protein undernutrition leads to profound and statistically significant immunological and functional alterations in the murine colon, which collectively impair the host’s capacity to respond to *Leishmania infantum* infection. In our model, a 21-day low-protein diet induced significant intestinal inflammation (elevated TNF-α, IL-17A, CCL5), microbiota dysbiosis (increased *Desulfobacterota* and *Firmicutes/Bacteroidota* ratio), elevated secretory IgA levels, and a disrupted cytokine profile, even in the absence of infection. When *L. infantum* infection was introduced in undernourished mice, these effects were exacerbated, resulting in intensified colonic inflammation (significant interaction effects for IFN-γ, IL-12, TGFβ, TNF-α, and IL-17A), enhanced dysbiosis, and a markedly impaired ability to control splenic parasite burden (*p* = 0.0098). These findings suggest that intestinal sensing of the parasite and the resulting immune responses are critically dependent on nutritional status, and that undernutrition severely disrupts gut-spleen immune communication, possibly through immune-endocrine pathways.

The observed shifts in the gut bacteriome, notably the enrichment of *Desulfobacterota* and *Firmicutes* and the depletion of *Bacteroidota*, *Alistipes*, and *Muribaculaceae*, reflect microbial adaptations to protein scarcity that may inadvertently promote inflammation, epithelial damage, and reduced antimicrobial signaling. The expansion of pathobionts such as *Enterobacteriaceae* was associated with higher levels of proinflammatory cytokines and correlates with previously reported histopathological damage in the gut.

**FIGURE 7:**
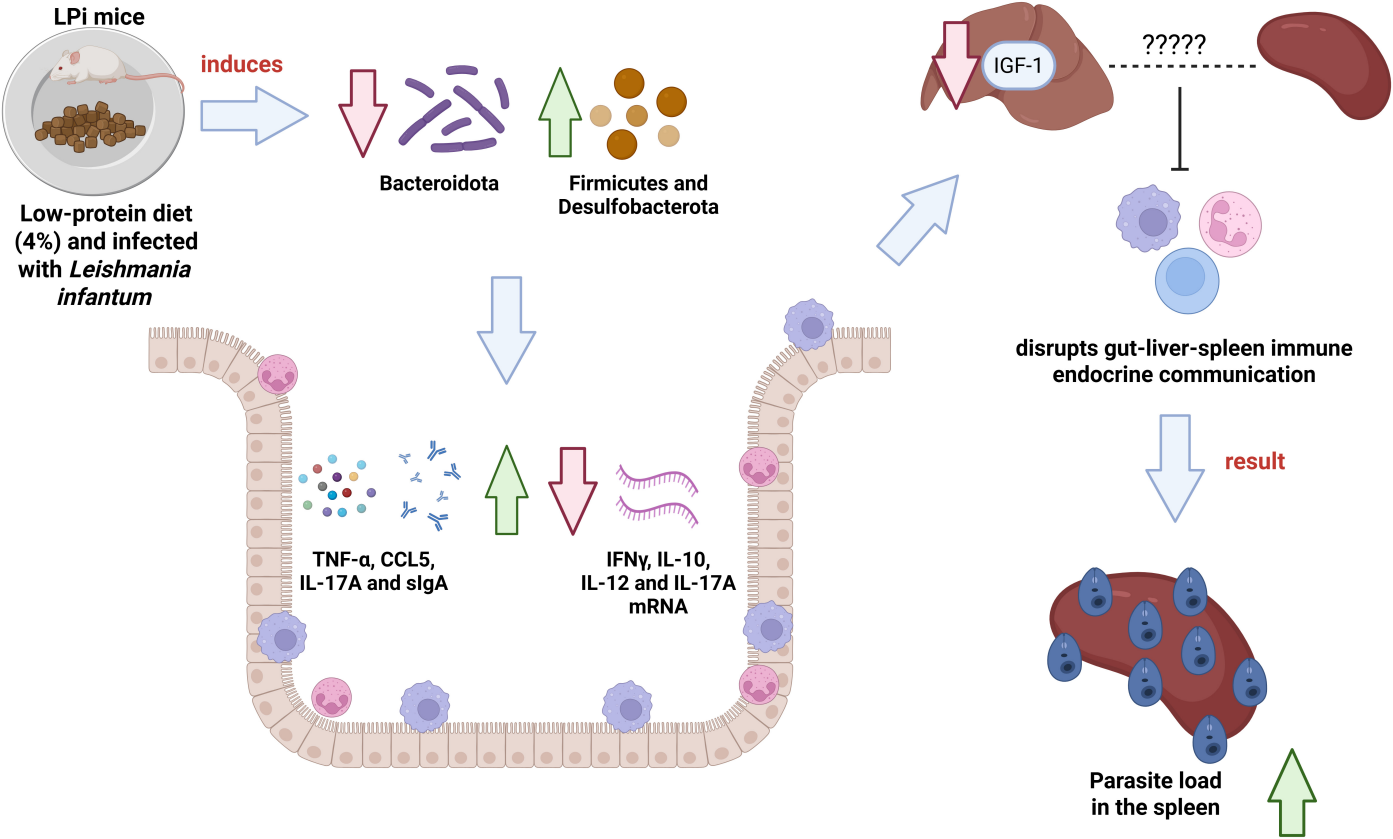
Proposed mechanistic pathway linking protein undernutrition to impaired control of *Leishmania infantum* infection. A low-protein diet (1) directly induces colonic dysbiosis, characterized by a decrease in *Bacteroidota* and an increase in *Desulfobacterota* and *Firmicutes* (2). This altered bacteriome triggers a state of local intestinal inflammation and immune disruption (3), evidenced by elevated luminal levels of pro-inflammatory cytokines (TNF-α, IL-17A, CCL5) and secretory IgA (sIgA), alongside tissue downregulation of cytokine mRNAs (IFN-γ, IL-10, IL-12, IL-17A). The resulting intestinal barrier dysfunction and inflammatory milieu are hypothesized to disrupt gut-liver-spleen immune-endocrine communication (e.g., via IL-13 and IGF-1 pathways) (4). This systemic immune dysregulation (5) ultimately compromises the host’s ability to control parasite replication in secondary lymphoid organs, leading to a significantly increased splenic parasite burden in undernourished and infected (LPi) mice (6).

A proposed mechanistic pathway integrating these findings is summarized in Figure 7. In brief, protein undernutrition initiates colonic dysbiosis, which drives local inflammation and immune imbalance. This disrupted intestinal environment impairs systemic immune-endocrine communication, ultimately compromising the host’s ability to control splenic parasite burden during *L. infantum* infection.

Taken together, our findings underscore that protein undernutrition not only compromises gut barrier function and microbial homeostasis but also disrupts systemic immune responses, facilitating the progression of visceral leishmaniasis. The statistical integration of biomarker data, from microbial phyla abundance and cytokine concentrations to organ weights and parasite loads, provides a coherent mechanistic narrative linking dietary protein deficiency to worse infection outcomes. These results highlight the critical need to consider nutritional interventions as an integral part of the clinical management and prevention of parasitic diseases in at-risk populations.

As future perspective, based on our findings, a critical next step will be to investigate the reversibility of undernutrition-induced dysbiosis and immune dysfunction through nutritional rehabilitation. Future studies will evaluate whether the dysbiotic profile, colonic histopathology, and associated inflammatory milieu in undernourished and infected mice can be restored to homeostasis after nutritional rehabilitation. Furthermore, we aim to determine if such dietary restoration can rescue systemic immunocompetence, thereby re-establishing control over splenic parasite burden. These experiments will clarify the plasticity of the gut-immune axis in the context of infection and could inform nutritional recovery strategies as a component of integrated disease management in vulnerable populations.

## Data Availability

The datasets presented in this study can be found in online repositories. The names of the repository/repositories and accession number(s) can be found in the article/Supplementary material.

## Footnotes

1
http://clioc.fiocruz.br/

2
https://www.microbiomeanalyst.ca/

3
https://www.ncbi.nlm.nih.gov/search/all/?term=PRJNA1262530
