# Supplementary material for: Protein undernutrition alters the colonic bacteriome, disrupts intestinal immune homeostasis, and impairs control of Leishmania infantum infection in a murine model of visceral leishmaniasis
Source: Front Nutr. 2026 Jan 30;12:1733703. doi: 10.3389/fnut.2025.1733703 (PMC12900761; doi:10.3389/fnut.2025.1733703)
Supplement: Supplementary file 1 [file Table_1.docx]

Supplementary Material

**Supplementary Table S1**. Sequences of primers used for real time qPCR

| **Accession**  **Number** | **Target** | **Forward Primer** | **Reverse Primer** | **Product size (bp)** | **Slope** | **Efficiency**  **(E) (%)** | **Correlation coefficient (R^2^)** |
| --- | --- | --- | --- | --- | --- | --- | --- |
| NM_010548.2 | ***IL-10*** | ACAACATACTGCTAACCGACTCCT | GGCAACCCAAGTAACCCTTAAAGT | 57 | 3.15 | 108 | 0.98 |
| NM_008351.3 | ***IL-12*** | CTCTCATATTCACTATACAAGTTG | GCTCTTCTGCTAACACAT | 94 | 3.18 | 106 | 0.95 |
| ﻿NM_010552.3 | ***IL-17A*** | ﻿TCTGTTCTCATCCAGCAAGA | ﻿ATCTTCTCGACCCTGAAAGT | 80 | 3.39 | 97 | 0.99 |
| NM_008337.3 | ***IFN-γ*** | AGGACACAACAAGATGGA | TTAGTGAGAGTGAACATTACAG | 141 | 3.30 | 100 | 0.92 |
| NM_001278601.1 | ***TNF-α*** | CTTCCTACCTTCAGACCTT | GCCTTCCAAATAAATACATTCAT | 153 | 2.60 | 120 | 0.97 |
| NM_011577.1 | ***TGF-β*** | ATTCCTGGCGTTACCTTGG | CCTGTATTCCGTCTCCTTGG | 117 | 2.46 | 155 | 0.85 |
| NM_025567.2 | ***Cyc1*** | GGTGTCATTGCGAGAAGG | GGTGCCATCATCATACTCC | 106 | 3.32 | 100 | 0.99 |
| NM_016774.3 | ***ATPβ5*** | TGAGTGTTGAGCAGGAGATTC | TTGGCGACATTGTTGATTAGC | 148 | 3.39 | 97 | 0.99 |
| NM_013556.2 | ***HRTP*** | CGTGATTAGCGATGATGAACC | AAGTCTTTCAGTCCTGTCCA | 124 | 3.00 | 107 | 0.99 |
| AF285161.1 | ***UBC*** | CTGTGAGAGCCGTGGATATTGG | GCACTTCCGTCTTTCAGCAAA | 84 | 3.47 | 94 | 0.98 |
| AF169140.1 | ***kDNA*** | TGTTGGTTGTGTGACTTTA | CATCCCACCAGACTAATC | 74 | 2.99 | 115 | 0.99 |
